# Barriers and Facilitators for Physical Activity in Adults with Type 2 Diabetes Mellitus: A Scoping Review

**DOI:** 10.3390/ijerph18105359

**Published:** 2021-05-18

**Authors:** Mireia Vilafranca Cartagena, Glòria Tort-Nasarre, Esther Rubinat Arnaldo

**Affiliations:** 1 Department of Nursing, Faculty of Health Science and Welfare, University of Vic-Central University of Catalonia (UVIC-UCC), Av. Universitaria 4-6, 08242 Manresa, Spain; mvilafranca@umanresa.cat; 2 Althaia Fundation, C/Dr Joan Soler 1-3, 08243 Manresa, Spain; 3 Department of Nursing, Faculty of Nursing and Physiotherapy, University of Lleida, C/Montserrat Roig, 25198 Lleida, Spain; esther.rubinat@udl.cat; 4 Health Education Research Group (GREpS), Faculty of Nursing and Physiotherapy, University of Lleida, Carrer de Montserrat Roig, 25198 Lleida, Spain; 5 Calaf Primary Care Center, Gerència Territorial Catalunya Central, Catalan Health Institute (ICS), Cta. Llarg19, 08280 Calaf, Spain; 6 Center for Biomedical Research on Diabetes and Associated Metabolic Diseases, Instituto de Salud Carlos III, 28220 Madrid, Spain

**Keywords:** Diabetes Mellitus type 2, exercise, motivation, patient compliance, healthy lifestyle, scoping review

## Abstract

The treatment of Type 2 Diabetes Mellitus (DM2) comprises physical activity (PA), diet, and medication. PA provides important benefits for people with diabetes. However, the majority of patients with DM2 do not attain the recommended levels of PA. Despite the evidence of the benefits to health of engaging in PA, the recommendations have not been fully translated into clinical improvements. Using a scoping review, this study aimed to identify the factors that influence levels of physical activity in adults with DM2. Eighteen studies published from 2009–2020 were identified by a search of relevant systematic databases between March 2019 and December 2020. The scoping review was carried out in accordance with the model defined by Arksey and O’Malley. The synthesis revelated sociodemographic characteristics, and six components—personal, motivation, social, mental, clinical, and self-efficacy—were identified as factors. Those that were most frequently identified were motivation and social support. In conclusion, these results should be considered to implement strategies to encourage people with DM2 to engage in physical exercise and thus improve the management of their condition.

## 1. Introduction

Due to its increasing prevalence and high burden of disease, Type 2 Diabetes Mellitus (DM2) is a significant global health problem. It is a major cause of blindness, kidney failure, heart attack, stroke, and lower limb amputation [1].

The International Diabetes Federation has reported that 463 million people had diabetes in 2019, of whom 90% to 95% were diagnosed with DM2. It is estimated that by 2045, 700 million will be diagnosed as diabetic [2].

The rise in the incidence of diabetes is due to the growing epidemic of overweight and obesity, sedentary lifestyles, and population aging [3]. DM2 carries a high economic burden [4].

It has been shown that the adoption of healthy life habits and adherence to medication reduce mortality and morbidity in chronic diseases such as DM2. However, the adherence to pharmacological treatments and the observance of healthy lifestyle habits in chronic diseases is currently estimated to be 50% [5], and previous studies have shown that long-term maintenance of weight loss and complete adherence to diet and recommendations to engage in physical exercise are rare in patients with DM2 [6,7,8].

The onset of DM2 forces patients to make a series of lifestyle modifications that have to be maintained permanently. Given the considerable effort required to modify deeply ingrained habits, it is clear that treatment is a challenge, particularly because adults are more resistant to change than younger age groups [5].

The treatment of DM2 comprises physical activity (PA), diet, and medication. PA provides benefits such as the improvement of insulin sensitivity and a reduction in body weight [9,10,11].

Physical inactivity is a major modifiable lifestyle risk factor associated with cardiovascular disease [12]. Among patients with DM2, however, the perceived need to increase PA is low (compared, for example, with improving dietary habits); with regard to PA, patients with DM2 present low levels of determination, action, maintenance, and stabilization-of-change [13,14]. Thus, the design of DM2 treatment protocols should seek to raise awareness of the importance of PA.

Most of the interventions aimed at promoting PA are focused on educational, informative, and directed aspects of nursing but do not consider aspects such as self-determination, motivation and social support that have been determined by the correct adherence to a new lifestyle [15,16,17].

Self-determination theory (SDT) is a general theory of human motivation that has been increasingly used to explain the internalization process that leads to autonomous motivation for permanent behavior change. In the most autonomous forms of motivation, exercise is performed because (a) it provides pleasure and satisfaction (intrinsic motivation); (b) the person integrates exercise as a central value in his/her value system (integrated regulation); or (c) the person simply values exercise as important for his/her health (identified regulation). Autonomous motivation is the strongest predictor of success in increasing PA. Physician–patient interactions and lifestyle interventions should focus on promoting self-motivated reasons for change and the integration of change within the personality [18].

The level of motivation for any change in behavior is known to be closely related to the probability of action. Once the motivation factor has been addressed, strategies can be introduced to manage competing priorities. This temporal ordering of intervention strategies is supported by the theory that people who lack motivation will be less receptive to discussing strategies for behavior change [19].

Autonomous or self-determined motivation is one of the factors that best predicts compliance with PA and is linked to factors of well-being. The concept of well-being comprises physical fitness and functional capacity, mental health and resources, and a positive attitude and quality of life [20].

Social support, defined as the structure and quality of social relationships, can improve health outcomes by improving adherence to healthy behaviors and by impacting emotions and mood [21]. Social support from both family and health personnel has been described as a medium-term facilitator: “Social support from family did not mediate short-term physical activity changes but was the most consistent mediator of intermediate-term changes of physical activity” [22].

There is growing evidence that patients’ motivation for effective self-management may be enhanced by an autonomous supportive health care climate [23]. Studies have found that autonomous or self-determined motivation for diabetes care predicts increased physical exercise [20,24].

Perceived autonomy support was associated with autonomous motivation, which increases the effect of perceived autonomy support on PA. Thus, autonomy support was associated with patients’ PA through autonomous motivation. Self-care competence did not mediate the effect of autonomous motivation on PA. Koponen [25] supports the idea of SDT that internalization of the value of good health behavior is necessary for engagement in a physically active lifestyle. Health care practitioners can promote patients’ PA by supporting their autonomous motivation. Awareness of the benefits of exercise does not predict action change because the physiological changes that individuals with diabetes can experience after exercise do not promote, directly, such a change. For this reason, interventions in this area should not only aim to raise awareness of the benefits of the new behavior, but also involve self-efficacy and social support in order to establish programs that allow control and maintenance of future health behaviours [26].

Despite the evidence of the benefits to health of engaging in PA, the recommendations have not been fully translated into clinical improvements. Adherence of patients with DM2 to PA remains low, despite the many attempts to increase it. Few studies published to date have sought to understand what drives patients with DM2 to adopt prevention behaviors and specifically PA.

## 2. Aim

This study aimed to systematically examine and synthetize published studies on the facilitators of, and barriers to, engaging in PA in adults with DM2. The identification of the main facilitators and barriers may inform the development of effective programs to increase and maintain PA levels of adults with DM2.

## 3. Methods Design

We performed a scoping review (SR) following the methodological model of Arksey and O’Malley [27] and the later adaptation by Levac et al. [28].

### 3.1. Identifying the Research Question

What are the factors that influence levels of physical activity in adults with Type 2 Diabetes Mellitus?

### 3.2. Identifying Relevant Studies

A search was carried out in the main bibliographic databases in the health sciences: SCOPUS, CINAHL, Cochrane, Web of Science (WOS), and PUBMED; and other secondary databases such as Open Access Theses and Dissertations (OATD). Additional sources were identified using other databases such as Google Scholar or free Internet searches.

The MeSH terms used were “Diabetes Mellitus Type 2, Exercise, physical activity, fitness, habits change, habits modification, habits choice, lifestyle change, compliance, motivation, barrier, facilitator, adherence, compliance, non-adherence, non-compliance”.

Table 1 describes the final search strategy, which was adapted to the selected databases according to the specific language used in each one (See Table 1).

Only papers published between 2009 and 2020 in Spanish or English were considered. The search period lasted from March 2019 to December 2020.

### 3.3. Study Selection

Quantitative, qualitative, and mixed designs were selected, plus different types of review (conceptual, narrative, or systematic). Participants were patients with DM2 who carried out some type of physical activity. Patients with Gestational Diabetes, Type 1 Diabetes Mellitus, and patients with prediabetes were excluded. Studies published between 2009 and 2020 in English or Spanish were admitted.

The results were exported to the Mendeley bibliographic manager database, version 1.19.3 (https.//www.mendeley.com, access on 22 January 2020). Duplicates were excluded. Subsequently, a peer review of title and abstract was performed, and then a peer review of the full text. Studies were excluded when they did not meet the inclusion criteria or did not focus sufficiently on the topic. MVC and GTN each selected a number of studies independently. Disagreements were resolved with the help of the third member of the research team (ERA).

The Excel 16.24 computer program (Microsoft, Washington, DC, USA) was used as the database and for the classification of the information. At this stage, the recommendations of Levac et al. [28] were followed to aid decision making among researchers. The entire research team held four meetings (one at the start, two during the course of the study, and one at the end), although the three reviewers involved maintained continuous contact throughout. This iterative process refined the search strategy and the inclusion of articles. 

### 3.4. Charting the Data

Applying an adaptation of the proposal of Sánchez-Meca [29], extrinsic data (author and year), methodological data (population and sample, design, evaluation and instruments, results) and substantive data (intervention or tolls, motivation or conditioning factors) in Table 1 were analyzed (see Table 2).

In this table the variables under study were recorded in two dimensions: either as barriers to PA, or as facilitators. These dimensions emerged inductively through the content analysis, which was first carried out independently by each researcher and later jointly displayed the main characteristics. 

### 3.5. Collating, Summarizing, and Reporting Results

The researchers followed the three steps recommended by Levac [28]: analysis, reporting the results, and considering the impact of the findings. A qualitative analysis was carried out, consisting of a report of the results according to the main characteristics of each study and type of review.

## 4. Results

### 4.1. Identification and Selection of Relevant Papers

**Figure 1:**
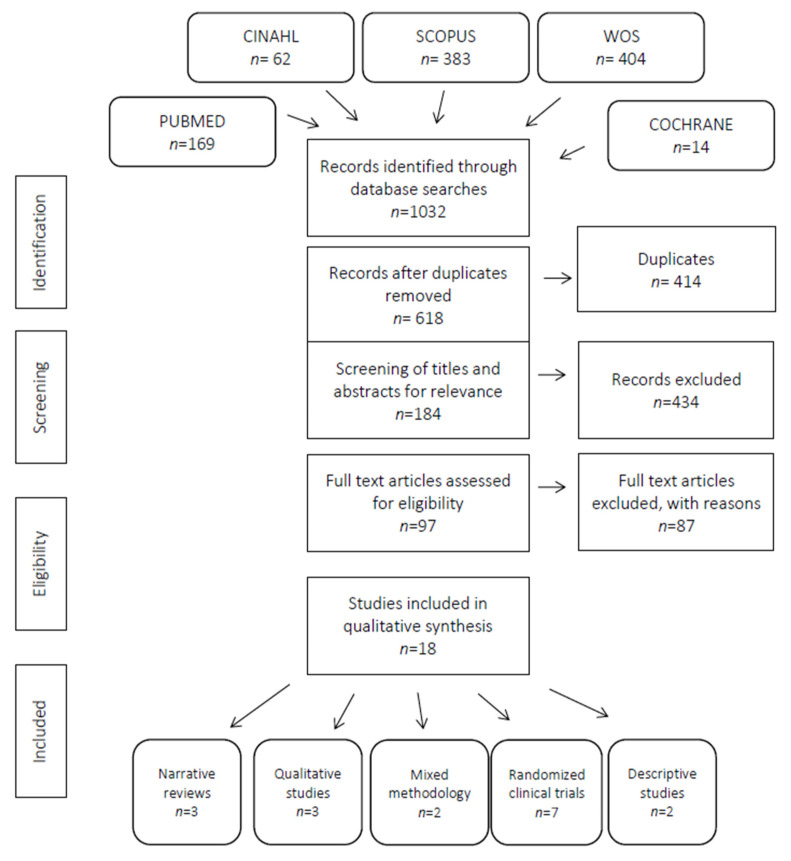
PRISMA flow-diagram of screening process for review.

A total of 1032 articles were obtained from the electronic databases. After removing 414 duplicates and excluding 434 articles after reviewing their titles and abstracts, the number of potentially eligible articles was reduced to 97. After the second phase of full-text screening, 87 further articles were excluded, leaving a final total of 18 articles. See Figure 1.

Of the 18 selected articles, six were from the USA [19,26,38,39,40,42], three from Italy [13,31,33], two from Australia [30,41], one from Portugal [33], one from the UK [30], one from Belgium [22], one from Canada [31], one from Denmark [41], one from Africa [40], and one from Finland [18]. All articles were published in English. Seven had a quantitative design, three a qualitative design, and three a descriptive design; there were three narrative reviews and also two mixed-method reviews.

### 4.2. Risk of Bias, Validity, and Methodological Quality

The methodological quality of the included studies was assessed using CASPE [43] (see Table S1). The total scores on this scale range from 0 (best) to 10 (worst). 

The articles analyzed here had scores ranging from 0 to 5. There were two very high-quality papers with scores of 0, indicating a high level of quality: [22] and [39]. There were also six very high-quality articles with scores of 1 [30,31,40,41,42] and [13] or 2 [18,31] and [32] and the quantitative part of the study by [30]. Studies with scores of 3 or 4 were classed as medium quality [39], the quantitative and qualitative parts of the mixed-methods review by [19,34,36,41]. Only two papers had scores of 5 and were therefore of medium–low quality: one observational [26] and the other the qualitative part of the mixed-methods review by [30]. The qualitative and quantitative parts of the two mixed-methods studies were analyzed separately.

### 4.3. Factors That Influence the Performance of Physical Activity in Adults with Type 2 Diabetes Mellitus

The synthesis shows the factors that were identified as facilitators of or barriers to the performance of physical activity in adults with DM2 in Table 2. The factors were classified according to socio-demographic characteristics and into six components: personal, motivation, social, mental, clinical, and self-efficacy (see Table 3).

Regarding socio-demographic characteristics, the key obstacles were older age coupled with longer disease duration [13,18], excess weight [30,33,41], and female sex. One study identified female sex as a facilitator because healthy behaviors were recorded more frequently among women [39] but another study identified being a woman as an obstacle, because opinions about women’s physical abilities may affect their self-efficacy to perform PA [30].

Regarding the personal component, the key factors identified as facilitators were having information about PA [35,36], higher educational level [13,33], wanting to be healthy [36], identification with acceptance of lifestyle [41], and achieving improvements in the control of DM2 [37,41]. Obstacles included poor knowledge and the perception that exercise potentially exacerbates illness [40], lack of time [30,33], fear of hypoglycemia [26,33], having to do moderate to high intensity exercise and having to overcome the initial challenge of exercising [41], lack of willpower and not being in the habit of exercising [33], and the low importance attributed to PA and the feeling of an inability to exercise [39].

Regarding the motivation component, the main facilitator was the motivation factor itself [18,19,30,31,35,41]. Other studies reported a lack of motivation as an obstacle [19,30,34].

With regard to the social component, the key factors identified as facilitators were social support from family and health professionals [19,22,31,34,37,39] and having a personal trainer [34,37]. Obstacles were the absence of social support [18,26,32,34,38], lack of recommendations or medical information [31,32,33,34], lack of information given by health professionals regarding the specific types of suitable PA, not knowing how often they should exercise or how to implement a plan for regular PA [33], having to do PA alone [30,32,33], and the lack of parks, gymnasiums, and other facilities nearby for carrying out PA [31,32,33].

Regarding the mental component, the only factor identified was depression, which was reported to be a barrier [30,39].

Regarding the clinical component, the key factors identified as obstacles were muscle and joint fatigue and pain [34,39]. 

With respect to the self-efficacy component, the self-efficacy factor itself was identified as a facilitator [18,26,30,42].

## 5. Discussion

### 5.1. Socio-Demographic Characteristics

The results of the study suggest that older subjects and women were less likely to engage in PA. The older the subjects, the less likely they were to perform PA, due to the possible associated complications, the physiological changes involved in the aging process, and the high prevalence of non-communicable chronic diseases, or a feeling of being unable to exercise [44,45,46]. In one study of patients with DM2, only 21% engaged in PA; 6% were normal weight individuals, 12% were overweight, and 3% were obese. Thus, obesity was found to reduce the likelihood of performing PA [44]. Women reported having less energy and less ability than men [47], and indicated they need to increase their confidence and overcome barriers in order to perform PA [48]. No studies identified female gender as a facilitator of PA with the exception of the study by [33]. 

### 5.2. Personal Component

Feelings of obligation, internal pressure, discomfort, and guilt do not favor adherence to PA. On the contrary, self-esteem, enjoyment, and a sense of challenge are the main incentives for discovering the benefits of PA [49]. The adoption of PA must be a gradual incremental process so as to allow adaptations at metabolic and functional levels. Individuals need encouragement and accompaniment; they need to be presented with new challenges that enable them to go beyond the current limits of their physical abilities [50]. For a change in behavior (such as an increase in PA) to have consistent, long-term effects, it is important that patients internalize it, identify with it, and acknowledge its importance [49].

### 5.3. Motivation Component 

Motivation was the factor most frequently identified as a facilitator of physical activity, and lack of motivation was described by several authors as a barrier. Motivation is a key factor in maintaining PA in the long-term [37]. Neel [50] described the syndrome of the emergence of genetic homeostasis due to prolonged periods of physical inactivity. This prolonged inaction can lead to a loss of physical fitness due to genetic dysfunction: not due to a gene deficiency, but to a lack of motivation to perform activities, which fails to stimulate proper functioning. Papín et al. [51] found a positive relationship between the motivation to perform PA and the time since the diagnosis of the disease, basic psychological needs (autonomy, competence, and social support), and respondents’ resilience. Furthermore, an inverse relationship was found between HbA1c and the motivation to perform PA and basic psychological needs [52].

### 5.4. Social Component

Social influence (exerted by sedentary friends or family members) also represents a disincentive for physical activity. Only 15% of the study population identified the lack of resources as an important limitation for carrying out PA [47]. This finding is consistent with previous research that highlights the importance of social support, but also its complex role in the management of diabetes self-care. For example, social support encompasses many behaviors such as giving advice, assistance, and listening [19]. According to the study by Alarcón-Moraa [53], patients with longer disease duration perceive less social support in general, and less emotional and affective support in particular. These results corroborate those of Kadirvelu [54], who mention that, although in most cases family support tends to be given freely, it tends to be more forthcoming when the diagnosis of diabetes is recent, and diminishes when the disease continues for a longer period. Regarding the lack of information, the peer training carried out at some centers has a positive impact on physical exercise, the use of health resources, and self-efficacy in care. It fosters a positive relationship between patients and health staff, generates group support and self-confidence, and facilitates emotional management [55]. 

### 5.5. Mental Component

The only mental component identified was depression. Twenty percent of adults with DM2 had depression, and women with DM2 were two or three times more likely to have depression than men with DM2 [56]. Depression in DM2 is associated with poorer glycemic control, poor self-control of diabetes, and a high risk of mortality [33]. Additionally, research has shown that antidepressant medication may have direct effects on glycemic control that are independent of its effects on weight and mood [57].

### 5.6. Clinical Component

Fatigue and muscle and joint pain were identified as barriers. Moderate to extreme pain was present in 57.8% of diabetic patients [58]. Pain was strongly associated with poor mental health and physical functioning, but not with poorer glycemic control. 

### 5.7. Self-Efficacy Component

Bandura conceptualizes self-efficacy as the confidence in one’s ability to carry out a specific behavior. Related to physical activity, self-efficacy refers to one’s confidence in being physically active in certain situations [59]. Those with higher self-efficacy were less likely to smoke and more likely to adherence to diet and engage in exercise [60]. Factors that predicted lower self-efficacy for exercise were lack of motivation, higher BMI, a diagnosis of coronary heart disease, more depressive symptoms, and female sex [30].

## 6. Strengths and Limitations of the Review

This scoping review compiled information from studies with a wide range of designs and methods, and was conducted with the rigor and transparency required [27]. It provides an overview of the available literature on the factors that favor or discourage the habit of physical activity in adults with DM2. However, possible limitations are the publication bias inherent in the literature, the lack of guidelines for carrying out specific methods, and the difficulty of summarizing the results of different studies. This review took a considerable amount of time to complete, due to the extensive search coverage required by the approach. There may be a risk of selection bias if not all the available data were identified.

## 7. Conclusions

This scoping review identified the main barriers and facilitators of the performance of physical activity in adults with DM2. Sociodemographic characteristics and six components—personal, motivation, social, mental, clinical, and self-efficacy—were identified as factors. Motivation and social support were the factors most frequently identified. Few studies published to date have explored the impact of these factors. It is important that future research should devise strategies for promoting adherence to PA that take into account the barriers and facilitators in populations with DM2 identified here in order to improve the effectiveness of intervention programs.

### Implications for Practice

This review can help health professionals and educators to reorient the focus of interventions and programs mainly dedicated to this topic. Effort should be made to create intervention tools that provide patients with DM2 with motivational engagement and encouragement of self-care.

Preventative measures and promotion of health would empower patients with DM2 with information, instruments, and strategies to obtain the most positive perspective, achieve better adherence to PA, and motivate them to adopt healthy behaviors and achieve maximum personal satisfaction.

## Figures and Tables

**Table 1:** Database search strategy.

1. exercise	11. barrier
2. physical activity	12. facilitator
3. fitness	13. adherence
4. 1 or 2 or 3	14. compliance
5. diabetes mellitus type 2	15. nonadherence
6. habits change	16. noncompliance
7. habits modification	17. motivation
8. habits choice	18. 11 or 12 or 13 or 14 or 15 or 16 or 17
9. lifestyle change	19. 4 and 5 and 10 and 18
10. 6 or 7 or 8 or 9	

**Table 2:** Characteristics of the studies’ design: quantitative, qualitative, and mixed.

Author(s), Year of Publication	Study Location	Study Population	Methodology	Theory	Intervention/Tolls (Web, Interview, Primary Care)	Motivation (Email, Motivational Interview)
[30] Alharbi, M., Gallagher, R., Neubeck, L., Bauman, A., et al. 2016	Australia	*n* = 134	Quantitative (randomized controlled trial) and qualitative (semi-structured interviews)	Bandura	1 h group-based supervised structured exercise twice a week and four 90 min group-based information sessions. Then, 3 telephone follow-up calls over the following 8 months	NO
[31] Balducci, S., Sacchetti, M., Haxhi, J., Orlando, G., Zanuso, S., et al. 2015	Italy	*n* = 300	Randomized controlled trial	Social cognitive theory and health belief model	Intervention in the INT group consisted of aggregated behavioral-change techniques once-a-year for 3 years. Theoretical, individual, face-to-face counseling sessions and practical exercise	Efforts are designed to convince the patient that regular PA is the pre-eminent cure for DM2 and to understand the positive expectations the individual patients had of this change in behavior
[32] Bekele, H., Asefa, A., Getachew, B., Belete, A.M.	Africa	-	Systematic Review	PICO. The levels of evidence and the quality guides of articles and research papers were evaluated based on the Johns Hopkins Method of Research Evidence Appraisal Tool.	No	No
[13] Centis, E., Trento, M., Dei Cas, A., et al. 2014	Italy	*n* = 1353 consecutive outpatients with DM2	Not specified. Use of questionnaires. Descriptive analysis	Prochaska’s model	Face-to-face questionnaire	Motivation to change was tested by the EMME-3 questionnaire for diet and PA
[30] Collins, T., Lunos, S., Ahluwalia, J. 2010	USA	*n* = 145 subjects with chronic disease (DM1 or DM2 or peripheral arterial disease)	Randomized clinical trial, cross-sectional study	Social Cognitive Theory	Questionnaires, treadmill walking, six-minute walk test	Walking intervention to improve distance at 6 months in individuals with DM2 and peripheral arterial disease
[33] Gallé, F., Di Onofrio, V., Cirella, A., et al. 2017	Italy	*n* = 130 Overweight and inactive patients with DM2	Neither experimental nor controlled. Pre-post, prospective	No	1-h training group sessions performed two times per week and short-form 12 questionnaire	Motivational program, a nutrition program, and an exercise program
[26] Gómez-Zúñiga, B., Pousada, M., Hernandez, M., et al. 2015	USA	*n* = 3916 people with diabetes completed the BBT (BIG BLUE TEST)	Not specified. RCT	No	Web site (shares his or her experience by collecting own data and answering some questions through the Web)	No
[18] Koponen, A.M., Simonsen, N., Suominen, S. 2018	Finland	*n* = 256	Observational, cross-sectional mail survey	Self-determination theory perspective	Interviews with successful and unsuccessful participants	Studied autonomous motivation but did not apply any interventions to increase it
[34] Laranjo, L., Neves, A., Costa, A., et al. 2015	Portugal	*n* = 16 Patients with type 2 DM were recruited at the Portuguese Diabetes Association outpatient clinic	Qualitative	No	Three video-recorded focus groups. Pre-tested interview guide	No
[35] Liebreich, T., Plotnikoff, R.C., Courneya, K.S., et al. 2009	Canada	*n* = 49	Prospective. 2-arm randomized controlled trial. Control/intervention groups	Social Cognitive Theory (SCT)	Web site	Individualized emails were sent on a weekly basis, providing general feedback on the specific topic of the week, progress, and motivation.
[19] Miller, S., Marolen, K. 2012	USA	*n* = 22 African women with DM2. Two focus groups of 11 participants	Qualitative	Trans-theoretical Model of Behavior Change	Focus group	No
[36] Patel, N., Ferrer Harriet, B., Tyrer, F., et al. 2017	UK	-	Narrative review	Emergent (‘berry picking’) model of information retrieval	No	No
[37] Schmidt, S.K., Hemmestad, L., Macdonald, C.S., Langberg, H., Valentiner, L.S.	Denmark	*n* = 6 (qualitative)	Longitudinal Qualitative Study	Health belief model (HBM), self-determination theory (SDT) and relevant research on the topic	Two rounds of in-depth, semi-structured interviews, conducted in August 2016 and February 2017	No
[38] Richardson, C.R., Buis, L.R., Janney, A.W., et al. 2010	USA	*n* = 324	2-arm randomized controlled trial	Bandura’s social-cognitive theory and social influence theories including social learning theory	Web page	Individually tailored motivational messages
[39] Schneider, K., Panza, E., Handschin, B., Ma, Y., et al. 2016	USA	*n* = 20	Randomization.	Linear mixed models: Littell, Stroup, Milliken, Wolfinger, and Schabenberger	Orientation session and 38 group exercise classes over 24 weeks	No
[40] Soderlund, P.D. 2018	USA	-	Review: type not specified	No	PRISMA	MI proficient counselors who emphasize that PA self-management may help foster PA behavior change
[22] Van Dyck, D., De Greef, K., Deforche, B., et al. 2011	Belgium	*n* = 143	Randomized controlled trial	Social cognitive theory of Bandura + Prochaska’s trans-theoretical model de and self-determination theory	Baseline questionnaire administered in patients’ homes by a psychologist (IPAQ). The subject was provided with a pedometer. Follow-up by telephone	There was no increase in autonomous motivation towards physical activity in this study group, although our intervention also incorporated self-determination theory constructs
[41] Wycherley, T., Mohr, P., Noakes, M., et al. 2012	Australia	*n* = 106 participants commenced and 84 completed the initial 16-week research based supervised lifestyle intervention program. Of the 81 participants invited, 3(37%) completed the 1-year follow-up	Not specified, but it was a non-randomized controlled trial and qualitative study	Standardized open-ended telephone interview	Getting involved in a structured exercise program may lead to improvements that may intrinsically motivate and facilitate exercise participation in the longer term	No

**Table 3:** Barriers to, and facilitators of, the performance of PA.

Author(s), Year of Publication	Conditioning Factors: Barriers	Conditioning Factors: Facilitators
[30] Alharbi, M., Gallagher, R., Neubeck, L., Bauman, A., et al. 2016	The most common barriers were lack of motivation (40.3%), lack of time overall (30.6%), and lack of time due to family commitments (17.2%). Baseline self-efficacy, depressive symptoms, being female, overweight, and having coronary heart disease	No
[31] Balducci, S., Sacchetti, M., Haxhi, J., Orlando, G., Zanuso, S., et al. 2015	Barriers that are outside the patient’s own control include lack of specific knowledge on the part of both physicians and exercise trainers and lack of dedicated facilities	No
[32] Bekele, H., Asefa, A., Getachew, B., Belete, A.M. 2020	Barriers included the poor knowledge, the perception that exercise potentially exacerbates illness, lack of an exercise partner, specific locations away from home, the rainy season in Africa, criticism by others, and lack of support from the partner, health professionals, family members, and friends. Lack of knowledge and education, poverty and cost, population changes, and lack of access to healthcare	No
[13] Centis, E., Trento, M., Dei Cas, A., et al. 2014	Older age and longer disease duration, Higher motivation to change was recorded in the area of diet compared to that of AF	Higher educational level, self-efficacy was higher in males
[42] Collins, T., Lunos, S., Ahluwalia, J. 2010	Walking alone or in rainy or cold weather	Self-efficacy, motivation
[33] Gallé, F., Di Onofrio, V., Cirella, A., et al. 2017	Physical barriers (disorders, excessive weight, hypoglycemic crisis), psychophysical barriers (laziness, lack of companions, of physician recommendations, low importance attributed to physical activity, feeling unable to exercise) and environmental barriers (lack of time, of green areas, of a gym close by, of equipment at home)	Personal trainer, higher educational level, women
[26] Gómez-Zúñiga, B., Pousada, M., Hernandez, M., et al. 2015	Less social support, DM1 fear of hypoglycemia	Social norms, self-efficacy
[18] Koponen, A.M., Simonsen, N., Suominen, S. 2018	Poor health, stress, and insulin medication slightly, higher age, poor health, and social support	Autonomous motivation, self-care competence and perceived autonomy support correlated
[34] Laranjo, L., Neves, A., Costa, A., et al. 2015	Lack of motivation and willpower, and not having created the habit of exercising. To a lesser degree: fatigue, muscle and joint pain, lack of information regarding the specific types of physical activities, lack of family or friend support. Themes PA: Decisional, Fatigue, Pain and Co-morbidities	Information and knowledge translation, as well as family and social ties
[35] Liebreich, T., Plotnikoff, R.C., Courneya, K.S., et al. 2009	No	Information accessed through the virtual library, and therefore increased their physical activity as well
[19] Miller, S., Marolen, K. 2012	Lack of motivation, laziness, competing priorities	Social support, motivation
[36] Patel, N., Ferrer Harriet, B., Tyrer, F., et al. 2017	Car travel, racial harassment, or abuse when exercising and, for women, expectations to remain in the home, fear for personal safety, lack of same gender venues and concerns over the acceptability of wearing ‘western’ exercise clothing	Weight gain might compromise family/carer responsibilities, desire to be healthy, DM2 diagnosis, and exercise classes held in ‘safe’ environments such as places of worship
[37] Schmidt, S.K., Hemmestad, L., Macdonald, C.S., Langberg, H., Valentiner, L.S. 2020	No	Five motivating factors were identified: achievement of results (reduce their daily medicine intake and live an overall healthier life), social support and relatedness (with help from the coaches, they developed skill and confidence in exercising), support from health care professionals and identification with acceptance of lifestyle (displayed signs of the new lifestyle being part of their lives and self-image)
[38] Richardson, C.R., Buis, L.R., Janney, A. W, et al. 2010	Participants with low baseline social support for physical activity used the online community features more than participants with high baseline social support	Participants in both arms who reported having social support at the end of the study were more likely to increase their step counts. More posts written, and pages viewed correlated with greater reported motivation to increase walking
[39] Schneider, K., Panza, E., Handschin, B., Ma, Y., et al. 2016	Pain, general exercise barriers and symptoms of major depression, comorbid depression and inadequately controlled diabetes	Family, social support
[40] Soderlund, P.D. 2018	Time within the healthcare setting	(1) counselors focused on a minimal number of DM2 self-management behaviors (2) MI counselors should emphasize either the frequency or duration of MI sessions (3) MI proficient counselors MI may be more effective when counselors prioritize a minimal number of target behaviors over the course of a few sessions
[22] Van Dyck, D., De Greef, K., Deforche, B., et al. 2011	Self-efficacy towards physical activity barriers was not a mediator during the intervention period (short-term), but only after the intervention ended (intermediate-term)	Positive social norms and modeling from family. Coping with relapse, defined as the ability to avoid and cope with relapse-inducing situations. Sport partner. Social support from family did not mediate short-term physical activity changes but was the most consistent mediator of intermediate-term changes of physical activity
[41] Wycherley, T., Mohr, P., Noakes, M., et al. 2012	Undertaking moderate to high intensity exercise and overcoming the initial challenge of doing exercise	Supervised exercise training during the program indicated access to appropriate programs/facilities, more affordable gym membership and having a personal trainer /motivator. the motivation derived from the general improvements they experienced during the program, encouragement and troubleshooting efforts of the staff, personal persistence and, less commonly, the motivating effects of having lost weight and achieved improvements in diabetes control. Support from staff

## Supplementary Materials

The following are available online at https://www.mdpi.com/article/10.3390/ijerph18105359/s1, Table S1: CASPE screening.
