# Supplementary material for: Barriers and Facilitators for Physical Activity in Adults with Type 2 Diabetes Mellitus: A Scoping Review
Source: Int J Environ Res Public Health. 2021 May 18;18(10):5359. doi: 10.3390/ijerph18105359 (PMC8157366; doi:10.3390/ijerph18105359)
Supplement: Supplementary file 1 [file ijerph-18-05359-s001.zip › ijerph-1163048-supplementary.pdf]

Table S: Articles analyzed with CASPE

Study details	Type of study	Is the trial guided by a clearly defined	Were patients randomly assigned to	Were all patients enrolled adequately	Was the blinding of the patients,	Were the groups similar at the start of	Apart from the intervention under	Does the treatment have a strong	What is the precision of this effect?	Can these results be applied in your	Were all the clinically important results	Do the benefits to be obtained justify	Number of points obtained
Liebreich et al. (2009)	RCT	0	0	1	1	0	0			0	0	0	1
Van Dyck et al. (2011)	RCT	0	0	0	0	0	0			0	0	0	0
Collins et al. (2010)	RCT	0	0	0	1	0	0			0	0	0	1
Gallé et al. (2017)	RCT	0	1	0	1	1	0			0	1	0	4
Richardson et al. 2010	RCT	0	0	0	1	0	0			0	0	0	1
Schneider et al. (2016)	RCT	0	0	0	0	0	0			0	0	0	0
Alharbi et al. (2016)	RCT	0	1	1	0	0	0			0	0	0	2
Balducci et al. (2015)	RCT	0	0	0	2	0	0			0	0	0	2
Wycherley et al. (2012)	RCT	0	1	1	0	0	1			0	0	0	3

**Study details**	**Type of study**	Does the study focus on a clearly defined	Was the cohort recruited in the most	Was the result measured accurately in order to	Did the authors take account of the power and	Was the follow-up of the subjects long enough,	What are the results of this study?	What is the precision of the result?	Do the results appear credible to you?	Do the results of this study coincide with other	Can the results be applied in your environment?	Will this change your clinical decision?	Number of points obtained
Koponen et al. (2018)	Observational	0	0	1	1	0			0	0	0	0	2

Centis et al. (2014)	Observational	0	0	0	1	0			0	0	0	0	1
Gómez-Zúñiga et al. (2015)	Observational	1	1	1	1	0			0	1	0	0	5

**Study details**	**Type of study**	Did the review focus on a clearly defined subject?	Did the authors look for the right kind of articles?	Do you think the most important and pertinent studies [illegible]	Do you think the authors of the review have made sufficient [illegible]	If the results of the different studies were mixed to obtain a combined result, was it reasonable to [illegible]	What is the overall result of the review?	What is the precision of the result/s?	Can the results be applied in your environment?	Were all the important results considered to make the [illegible]	Do the benefits outweigh the costs?	Number of points obtained
Soderlund (2018)	Review	1	0	0	1	0			1	0	0	2
Patel et al. (2017)	Review	0	0	2	0	0			1	0	0	3

**Study details**	**Type of study**	Were the aims of the study clearly defined?	Is the qualitative methodology appropriate?	Is the research method suitable for meeting the [illegible]	Was the strategy for selecting participant consistent [illegible]	Were the data collection techniques consistent with [illegible]	Was there a reflection on the relationship between [illegible]	Were ethical aspects taken into account?	Was the data analysis sufficiently rigorous?	Is the presentation of the results clear?	Are the research results applicable?	Number of points obtained

Laranjo et al. (2015)	Qualitative	0	0	0	0	0	2	0	1	0	0	3
Miller et al. (2012)	Qualitative	0	0	0	0	0	2	0	0	0	1	3
Alharbi et al. (2016)	Qualitative	0	0	0	2	0	2	0	1	0	0	5
Wycherley et al. (2012)	Qualitative	0	0	0	1	0	2	0	1	0	0	4
